# Molecular characterization of two hantavirus strains from different rattus species in Singapore

**DOI:** 10.1186/1743-422X-7-15

**Published:** 2010-01-22

**Authors:** Patrik Johansson, Grace Yap, Hwee-Teng Low, Chern-Chiang Siew, Relus Kek, Lee-Ching Ng, Göran Bucht

**Affiliations:** 1 DSO National Laboratories, 20 Science Park Drive, 118230, Singapore; 2 Environmental Health Institute, 11 Biopolis Way, #06-05/08, 138667, Singapore

## Abstract

**Background:**

Hantaviruses cause human disease in endemic regions around the world. Outbreaks of hantaviral diseases have been associated with changes in rodent population density and adaptation to human settlements leading to their proliferation in close proximity to human dwellings. In a parallel study initiated to determine the prevalence of pathogens in Singapore's wild rodent population, 1206 rodents were trapped and screened. The findings established a hantavirus seroprevalence of 34%. This paper describes the molecular characterization of hantaviruses from *Rattus norvegicus *and *Rattus tanezumi*, the predominant rodents caught in urban Singapore.

**Methodology:**

Pan-hanta RT-PCR performed on samples of *Rattus norvegicus *and *Rattus tanezumi *indicated that 27 (2.24%) of the animals were positive. sequence analysis of the S and M segments established that two different hantavirus strains circulate in the rodent population of Singapore. Notably, the hantavirus strains found in *Rattus norvegicus *clusters with other Asian Seoul virus sequences, while the virus strains found in *Rattus tanezumi *had the highest sequence similarity to the Serang virus from *Rattus tanezumi *in Indonesia, followed by Cambodian hantavirus isolates and the Thailand virus isolated from *Bandicota indica*.

**Conclusions:**

Sequence analysis of the S and M segments of hantavirus strains found in *Rattus norvegicus *(Seoul virus strain Singapore) and *Rattus tanezumi *(Serang virus strain Jurong TJK/06) revealed that two genetically different hantavirus strains were found in rodents of Singapore. Evidently, together with Serang, Cambodian and Thailand virus the Jurong virus forms a distinct phylogroup. Interestingly, these highly similar virus strains have been identified in different rodent hosts. Further studies are underway to analyze the public health significance of finding hantavirus strains in Singapore rodents.

## Background

The *hantavirus *genus in the *Bunyaviridae *family contains several important human pathogens that are prevalent worldwide. This group of viruses includes the etiological agents of hemorrhagic fever with renal syndrome (HFRS), largely seen in Europe and Asia, and hantaviruses causing (cardio) pulmonary syndrome (HCPS) in the Americas. The clinical severity of hantavirus infections ranges from asymptomatic infections to fulminate hemorrhagic shock and death. Hantaan virus (HTNV) and Dobrava viruses (DOBV) are causative agents of severe forms of HFRS and mortality rates of up to 15% have been reported. About 20 - 30% of HTNV infected patients develop hemorrhages [1,2].

Hantaviruses are enveloped and contain genomes composed of three negative-stranded RNA segments; small (S), medium (M) and large (L) segment, named according to the size of the individual RNAs [3]. The L segment encodes the viral RNA dependent RNA polymerase (RdRp), whereas M and S segments encode for the two envelope proteins (Gn and Gc) and the nucleocapsid protein (N), respectively.

Transmission of hantavirus to humans occur mainly through inhalation of aerosolized rodent excreta and hantavirus infections are therefore limited to the geographic regions inhabited by the infected animal hosts. Today, a wide array of hantaviruses has been detected in numerous rodent or insectivore species [4-6].

Hantaviruses are endemic in many countries of the world, and the trend in recent years indicates that the natural foci have extended from rural to more urban areas. The mainland China accounts for the majority of all cases reported worldwide and HTNV and Seoul virus (SEOV) are known to be the most prevalent causative agents of HFRS in Asia [7-9]. Specimens collected from 22 laboratories in China confirmed SEOV in 7 of 22 HFRS patients [7]. Furthermore, when comparing the nucleotide sequences of viruses from HFRS patients and rats captured in Beijing area, a nucleotide sequence identity of 96.3% to 99.7% was observed, indicating that SEOV is an important and perhaps the most common hantavirus in China [7]. Interestingly, additional rodent hosts and new hantaviruses are frequently discovered in Asia including Thailand virus, isolated from the great bandicoot rat, *Bandicota (B.) indica *in 1994 [10-12], and hantavirus genetic material extracted from *Rattus (R.) tanezumi *and *R. norvegicus *in Indonesia [13-15].

Many *Rattus *species are difficult to distinguish morphologically and there are many changes over the years in both genus and assignments [16]. Recently the barcoding technique has been proposed as a method for the identification of species on the bases of evolutionary divergence of a gene region such as mtDNA *cytochrome oxidase I *[17] or the *cytochrome b *gene [18]. According to Musser & Charlton 2005, seven groups of rodents are recognized within the Rattus genus including the *R. rattus *group and the *R. norvegicus *[19], and the *R. rattus *group comprises about 21 species including *R. rattus *and *R. tanezumi*. The *R. rattus *species is further divided into two subspecies based on the chromosome number [20]: an Ocean/European variant which Musser & Charlton named *R. rattus *and an Asian type that was named *R. tanezumi*.

In the late 80's in Singapore, Wong and co-workers found evidence of hantavirus infections in both rodents and humans and one hantavirus strain (R36) was isolated from *R. norvegicus *[21]. They also analyzed the seroprevalence in patients and found that 8.3% of Dengue Hemorrhagic Fever (DHF) suspects were seropositive to hantaviruses. However, for the last 15 years, only one case with classical manifestations of HFRS, confirmed by serology, have been reported in Singapore [22]. In a parallel study initiated to determine the seroprevalence of rodent-borne pathogens in Singapore's wild rodent population, 1206 rodents were trapped and screened (unpublished data). Findings in that study indicate that one-third of *R. norvegicus *and one-fifth of the *R. tanezumi *rodents were seropositive towards the SEOV nucleocapsid protein. Of the seropositive rodents, 5.5 and 26% were also tested positive by PCR, respectively.

This paper describes the subsequent screening of the animals using PCR, and the characterization of two genetically different hantavirus strains denoted Seoul virus strain Singapore and Serang virus strain Jurong TJK/06 of samples from *R. norvegicus *and *R. tanezumi*, respectively. Phylogenetic analysis, nucleotide and amino acid sequence identity were also determined.

## Materials and methods

### Ethics Statement

All animals were handled in strict accordance with good animal practice, as defined by The Animal Research Ethics Committee of DSO National Laboratories, 20 Science Park Drive, Singapore 118230.

### Rodent identification

The rodents were identified through PCR based amplification and sequencing of *cytochrome b *gene fragments obtained by using the primers mcytb1 and mcytHb (Ken Aplin, personal communication) before phylogenetic-based species identification was conducted. Obtained rodent sequences are available from the authors or accessible from GenBank (GQ274946 - GQ274949).

### Serology

Indirect Enzyme-linked immunosorbent assay (ELISA) was performed using a recombinant truncated nucleocapsid protein of the Seoul virus (M34881) and serum samples of collected rodents. The analysis was done as described earlier [23]. The calculated cut-off value (0.135) was set as 3 times the value of sera derived from negative lab. rats (*R. norvegicus*).

### Design of primers for Pan-hantavirus RT-PCR

Primers with the potential to detect all known rodent-borne hantaviruses were designed towards conserved regions identified from multiple alignments of hantavirus L segment sequences by the BioEdit program package [24]. Using the primer pair HantaL_2_fwd_3312-40 5'- TYTTTGARTTTGCHCAYCAYTCWGATGATGC and HantaL_2_rev_3481-53 5' - TCATGNARRTTRAACATRCTYTTCCACA for PCR, tissue samples (lungs and kidneys) of all rodents were analyzed, as described below. The PCR was done in Mg^++ ^free PCR buffer supplemented with 2 mM of MgCl_2_, 0.2 mM of dNTP, 1 μM of the primers and 1.25 U of AmpliTaq^® ^(Applied Biosystems Inc., USA), in a total volume of 25 μl. After a preincubation for 5 min at 95°C, 35 cycles at 53°C for 30 sec, 72°C for 45 sec and 45 sec at 95°C were done. After a final incubation for 10 min at 72°C the result was examined by agarose gel electrophoresis. A resulting PCR product of 170 bp was considered PCR positive.

### Extraction of RNA, reverse transcription and sequencing

RNA was extracted from tissue samples of kidneys and/or lungs of individual rodents using Qiagen RNeasy Mini kit (Qiagen, Inc., Germany) or the TRIzol reagent (Invitrogen Inc., USA), according to manufacturer's instructions. Reverse transcription of RNA was performed with SuperScript III (Invitrogen Inc., USA) and random hexamers. For direct sequencing of overlapping amplimers, numerous PCR primers were constructed using the PriFi software [25]. Specific cDNA fragments obtained by PCR were purified using QIAquick Gel Extraction kit or QIAquick PCR Purification Kit (Qiagen, Inc., Germany) before sequencing was conducted. Thereafter, the sequences of the segments were confirmed using a new set of primers targeting the determined cDNA sequences. However, the 15-20 nucleotides at the 5' and 3' termini of the S and M segments were set by the primers used for PCR. Finally, the resulting sequence data was assembled as chromatograms, using SeqMan (LaserGene Inc., USA) and edited with the BioEdit software package [24]. With the exception of the conserved 3' and 5' pan-handle sequence the S and M and partial L sequences of these hantavirus strains of Singapore have been made available (Genbank accession no's: GQ274936 - GQ274945).

### Phylogenetic analysis

The resulting sequences were aligned by ClustalW [26], as implemented in the BioEdit software package before bootstrapped maximum parsimony and trees were calculated using the SeqBoot, DNApars (setting: search for best tree, more thorough search, use input order, multiple data sets 1000 and 10 jumbles) and Consence softwares. Branch lengths of the trees were calculated using DNAml of the Phylip 3.67 software package as distributed by the author [27]. The tree was visualized by the TreeView software [28]. The similarity plots were performed by Stuart Ray's SimPlot 3.5.1 software using a window of 200 bp and steps of 20 bp, GapStrip: on, Kimura (2-parameter), T/t: 2.0 [29].

## Results

### Characterization of the rodent samples

The phylogeny-based analysis of the *cytochrome b *gene identifies the rodent hosts of the Seoul virus strain Singapore (RN41 and 46) as *R. norvegicus*. Furthermore, the rodent hosts carrying the virus strain Jurong TJK/06 (RT49 and 50), (in this paper denoted *R. tanezumi*) clearly clusters with *R. rattus diardi*, *R. tanezumi *and *R. kandianus *of the *diardii *clade of the R. rattus complex (Additional file 1, Figure S1) as described by Robins et.al [18].

### Serological and RT-PCR screening of rodent samples

**Figure 1:**
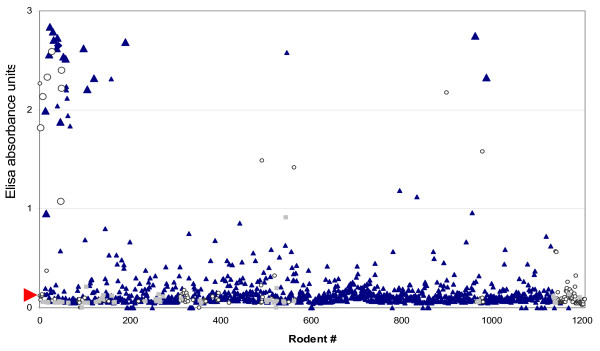
**Hantavirus positive rodents**. Scatter plot displaying along the horizontal axis the individual rodents captured in Singapore between 2006-2008 and along the vertical axis, absorbance units determined by ELISA using serum samples of indicated rodents. Dark blue triangles indicate *R. norvegicus *and open circles *R. tanezumi*. Other rodent species are pointed out as grey squares. Individual dots are shown as Large or Small data points indicating PCR positive or negative rodents, respectively. The calculated cut-off value (0.135) is indicated by a red arrow.

RT-PCR screening of kidney samples from rodents with Pan-hantavirus primers for the L segment, together with a succeeding PCR targeting the S segment revealed that 2.1% (21/996) of *R. norvegicus *and 4.5% (7/156) of *R. tanezumi *were PCR positive for hantavirus RNA. An observation was made when comparing the optical density OD values of the ELISA with the PCR results. It was noticed that rodents with the highest OD values were more likely to be PCR positive too. Among the rodents with OD values more than 1.0, 70% (7/10) were also found PCR positive (Fig 1). In contrast, none of the 17 *R. tanezumi *and only 2 of the 335 seropositive *R. norvegicus *having OD values less than 1.0 were also PCR positive. The exceptions included one rodent with a slightly lower ELISA OD value (0.96) and one ELISA negative but PCR positive *R. norvegicus*. It is likely that the latter rodent was infected close to the time of trapping.

### Sequence analysis

For hantavirus sequence analysis, two PCR positive lung samples of each of the two rodent species (*R. norvegicus *and *R. tanezumi*) were selected: rodent # 41, 46 and 49, 50, respectively. When comparing the complete open reading frames of the S segments from the two rodent species of Singapore with other complete or partial hantavirus S segment sequences (Additional file 2, Table S1), the hantavirus of the Singaporean *R. tanezumi *(strain Jurong) showed the highest nucleotide sequence identity (95.0%) to the Serang virus, obtained of *R. tanezumi *from Indonesia [13]. In addition, the Jurong sequence also demonstrated a high nucleotide sequence identity to partial sequences of the S segment of rodent samples of Cambodia (85.8%) [11] and full length S sequences of the Thailand virus (83.6%), isolated from *B. indica *[30].

Sequence comparisons of M segments also revealed the Serang virus as most similar (94.5%) to the Jurong strain. However, M and L segment of the Cambodian hantavirus strains are not available in public databases and only 343 bp and 412 bp of the Serang virus are currently accessible for sequence comparisons. As shown in Additional file 2, Table S1, analyses of nucleotide and amino acid sequences of the S and M segments strongly suggest that the Serang and Cambodian isolates, together with the Thailand virus share the closest identity to the Jurong virus strains of Singapore. When comparing the L segment sequences of the Jurong strains with the Serang virus, a nucleotide sequence identity of 91.2% was found, slightly lower than between the corresponding S or M segments. Though, the deduced amino acid sequences of the S segment encoding N, the M segment encoding Gn and Gc and the L segments encoding RdRp are nearly identical over the overlapping regions.

An analogous high nucleotide sequence identity was also noticed when comparing hantavirus S segment sequences of hantaviruses of *R. norvegicus*. The nucleotide sequence identity between hantavirus strains of Seoul Singapore and Cambodian Seoul virus strains was found to be between 97 and 99% and the highest identity was observed towards the Cambodian strain 41 (Camb 41) with 99.1% identity over the overlapping S segment sequences.

### Results of similarity plot (SimPlot)

**Figure 2:**
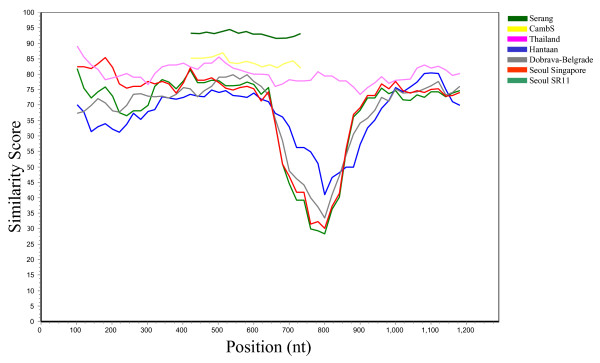
**Nucleotide sequence identity by SimPlot comparison**. SimPlot analysis of selected hantavirus genes encoding the nucleocapsid protein. The nucleotide sequences were analyzed in a window of 200 nucleotides and steps of 20 nucleotides. The complete open reading frames of the Singaporean hantavirus strain Jurong TJK/06 (This study) were chosen as query and Seoul SR11 (M34881), Hantaan virus (AB027111), Dobrava-Belgrade (L41916), Seoul Singapore (This study) and Thailand virus (AM397664) as references. SimPlot comparison of the partial sequences of the Serang virus (AM998808) and Cambodian strains (AJ427511) are shown in dark green and yellow curves in addition to the above sequences. Before analysis these two sequences were truncated at the 5' and 3' ends into a 486 nt overlapping region of the Serang virus and the Camb117 and Camb132 strains and compared against the corresponding Jurong TJK/06 cDNA sequence.

SimPlot comparisons of the complete open reading frames of the Jurong virus with the Seoul/Hantaan/Dobrava types of viruses demonstrated a significant non-conserved region of approximately 300 nt near the middle of the S segment [29]. However, such drop in nucleotide sequence identity was not noticed towards the Thailand virus sequences or against partial nucleotide sequences of the corresponding Cambodian strains or towards the Indonesian Serang virus (Fig 2). Moreover, comparisons of the amino acid sequences revealed a similar trend, although the decrease in similarity in the non-conserved region was less prominent (data not shown).

Due to the lack of available sequences in public databases, a comprehensive SimPlot comparison of the M segment could only be performed towards the Thailand virus along with a subset of less related hantaviruses. As expected, the M segment of the Jurong strain was most similar to the Thailand virus, especially when comparing the amino acid sequence of the glycoprotein Gn. In addition, two highly conserved regions consisting of approximately 300 amino acids each were found near the middle of the glycoproteins of the Jurong and Thailand viruses (data not shown).

### Phylogenetic analysis

**Figure 3:**
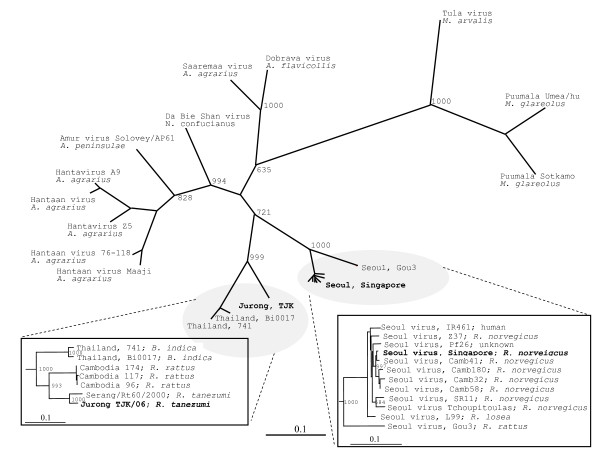
**Phylogenetic trees of hantavirus S sequences**. Phylogenetic trees of hantaviruses based on the complete open reading frames (upper top figure) and partial S segment sequences (bottom left and right figures). In trees, the results after 1000 bootstrap replicates are indicated. (Upper top figure), Tula virus (Z69991); Puumala virus Umea/hu AY526219); Puumala Sotkamo (X61035); Dobrava-Belgrade virus (L41916); Dobrava/Saaremaa/160v (AJ009773); Da Bie Shan virus NC167 (AB027523); Amur virus Solovey/AP61/1999 (AB071183); Hantaan virus A9 (AF329390); Hantaan virus Hu (AB027111); Hantavirus Z5 (EF103195); Hantavirus 76-118 (AF321095); Seoul virus IR461 (AF329388); Hantaan Maaji (M14626); Jurong TJK/06 (this study); Thailand virus (Nakhon Ratchasima/Bi0017/2004) (AM397664); Thailand virus 741 (AB186420); Seoul virus Gou3 (AF184988); Seoul virus Singapore (this study). (Bottom left) Thailand virus (Nakhon Ratchasima/Bi0017/2004) (AM397664); Thailand virus 741 (AB186420); Cambodia 96 (AJ427512); Cambodia 174 (AJ427513); Cambodia 117 (AJ427511); Serang virus/Rt60/2000 (AM998808); Jurong TJK/06 (This study). (Bottom right) Seoul virus IR461 (AF329388); Seoul virus Z37 (AF187082); Seoul virus Pf26 (AY006465); Seoul virus Singapore (this study); Seoul virus Camb41 (AJ427501); Seoul virus Camb180 (AJ427506); Seoul virus Camb32 (AJ427508); Seoul virus Camb58 (AJ427510); Seoul virus SR11 (M34881); Seoul virus Tchoupitoulas (AF329389); Seoul virus L99 (AF288299); Seoul virus Gou3 (AF184988).

Phylogenetic analysis of S segment sequences showed that the Jurong virus strains and the Thailand virus isolates cluster together (Fig 3). Other hantaviruses carried by rodents of the *Cricetidae *family, *Arvicolinae*, *Neotominae *or *Sigmodontinae *subfamilies (e.g. Puumala virus, Tula virus, Sin Nombre and Andes virus) or hantaviruses carried by family *Muridae *subfamily *Murinae*, such as Seoul virus, Hantaan virus and Dobrava-Belgrad virus are genetically more distant (Fig 3). When partial sequences were included to the phylogenetic analysis, a novel clade consisting of the Thailand, Jurong, Serang and Cambodian hantaviruses was found. According to the phylogenetic trees and distance matrix, these sequences are clearly strains of the same hantavirus species. A common origin seems evident in such case.

**Figure 4:**
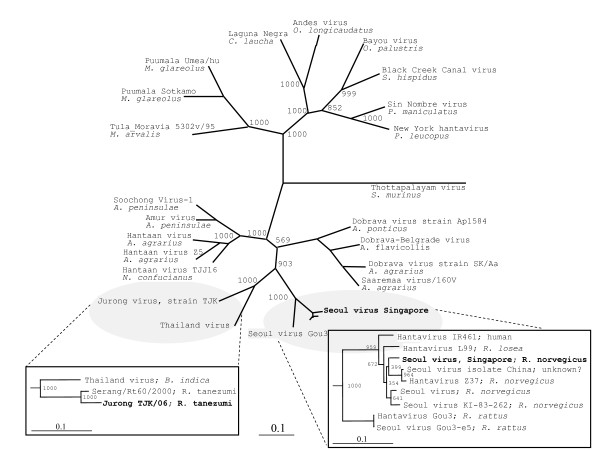
**Phylogenetic trees of hantavirus M sequences**. Phylogenetic trees of hantaviruses based on the complete open reading frames (upper top figure) and partial M segment sequences (bottom left and right figures). In the trees, the results after 1000 bootstrap replicates are indicated. (Upper top figure) Thottapalayam VRC-66412 (EU001329); New York hantavirus (U36802); Sin Nombre virus (NC_005215); Black Creek Canal virus (L39950); Bayou virus (L36930); Andes virus (NC_003467); Laguna Negra virus (AF005728); Puumala virus Umea/hu (AY526218); Puumala Sotkamo (NC_005223); Tula_Moravia 5302v/95 (NC_005228); Soochong virus SC-1 (AY675353); Amur virus JilinAP06 (EF371454); Hantaan virus (NC_005219); Hantaan virus Z5 (EU074224); Hantaan virus TJJ16 (EU074672); Jurong TJK/06 (This study); Thailand virus (L08756); Hantavirus Gou3 (AF145977); Seoul virus, Singapore (this study); Dobrava virus DOB/Saaremaa/160V (AJ009774); Dobrava virus SK/Aa (AY961616); Dobrava-Belgrade virus (L33685); Dobrava-Belgrade virus Ap1584/Sochi-01 (EU188450). (Bottom left) Thailand virus (L08756); Serang virus Rt60/2000 (AM998807); Jurong TJK/06 (This study). (Bottom right) Hantavirus IR461 (AF458104); Hantavirus L99 (AF288298); Seoul virus, Singapore (this study); Seoul virus China (EU163437); Hantavirus Z37 (AF187081); Seoul virus (NC_005237); Seoul virus KI-83-262 (D17592); Hantavirus Gou3 (AF145977); Seoul virus Gou3-e5 (AF288650).

As indicated by the phylogenetic trees for the viral S and M segments (Fig 3 and 4, respectively) the Jurong virus strains discovered in Singapore rats is genetically similar and form a clade with the Serang virus of Indonesia, Cambodian strains (e.g. Camb174, Camb117, Camb96) and hantavirus sequences of *B. indica *from Thailand (Thailand virus). Interestingly, the four closely related viruses; Jurong, Cambodia, Serang, and Thailand are carried by two or more different rodent species. Hantavirus strains of *R. norvegicus*, such as the Seoul Singapore and corresponding Cambodian isolates (e.g. Camb32, 41, 58 and 180) cluster closely together with other Seoul virus sequences, see Fig 3. The closest identity of the Seoul Singapore strain was observed towards the Cambodian strain 41 (Camb 41) with 99.1% identity for the overlapping S segment sequences.

## Discussion

There are increasing numbers of studies from Asia reporting prevalence of hantaviruses in rodent and human populations, suggesting emerging hantaviral infections in this part of the world [31]. Moreover, studies conducted, particularly in Southeast Asia, indicate that hantavirus diversity is expanding [8]. Under these circumstances, we initiated a series of studies that aimed to provide a clearer perspective of the hantavirus situation in Singapore, as well as other rodent-borne pathogens (on going study).

Due to difficulties in the morphological identification of rodent species, genetic barcoding may be used as an additional help for rodent classification. By comparing genes such as mtDNA *cytochrome oxidase I *or the *cytochrome b *gene, the rodent hosts of the Seoul virus Singapore strains and the viruses of the Jurong strain were identified as rodents of the *R. norvegicus *and rodents of the *R. diardii *clade, respectively. An interesting point to note is that rats identified as *R. tanezumi *are found within three different clades in the paper published by Robins et al, 2007 [18]. The three clades are the monospecific tanezumi clade, the tiomanicus clade and the diardii clade together with *R. rattus diardi *and *R. kandianus*, members of which came from Sri Lanka, Peninsular Malaysia, Java and northern Sulawesi (Additional file 1, Figure S1). Evidently, *R. rattus diardi*, *R. kandianus *and *R tanezumi *sequences are so similar, when found in the diardii clade, indicating that they are probably representatives of the same species.

In this study, we have identified and characterized two hantaviruses from the lungs of *R. tanezumi *of the diardii clade or R. rattus linage IV (Ken Aplin, personal communication) and *R. norvegicus*. The viral strains were denoted Jurong TJK/06 (RT49 and 50) and Seoul Singapore (RN41 and 46), respectively. Nucleotide sequences of the S and M segments as well as partial L cDNA sequences confirmed that these two virus strains are genetically distinct from each other. The nucleotide sequence identity of the S and M segments between the Jurong and Seoul Singapore strains was only 78% and 75%, whereas the amino acid sequence identity were 87% and 83%, respectively. These levels are much lower than the officially accepted 93% amino acid identity required for defining hantaviruses as same serotypes, or 7% divergence required for defining hantaviruses as different serotypes [3]. Nevertheless, factors such as the specific rodent hosts, distinct genetic lineage and possible reproductive isolation evident from the coexistence of these viruses within the same geographic regions further support our view that these virus strains are distinct from each other.

Phylogenetic analysis of hantaviruses of the Asian region revealed a close genetic relationship of the Jurong virus with the Thailand hantavirus carried by *B. indica *[30] and Cambodian hantavirus strains [11]. By analyzing recently submitted hantavirus sequences from Indonesia we noticed that the Serang virus, from the *R. tanezumi *[13] showed the highest nucleotide sequence identity with regard to the S (95%) and M (94.5%) segments of the Jurong virus sequence. Furthermore, the corresponding amino acid sequence of the L segment was found to be nearly identical over an overlapping region of 137 amino acids (aa 973-1109), while the corresponding nucleotide sequence identity over the same region was only 91.2%.

SimPlot analysis of S segment sequences from genetically and geographically different hantaviruses revealed a highly variable region in the middle of the S segment. A similar observation was made by Schmaljohn *et al *[3] after comparing the amino acid residues 240-310 of the nucleocapsid proteins of HTNV, SEOV and PUUV. In contrast, this region is highly conserved between the Jurong strains and the Thailand virus. Our analysis of available partial sequences of the Cambodian and the Serang virus S segments suggests that these hantaviruses are also likely to share the same characteristics (Additional file 2, Table S1).

SimPlot analysis of open reading frames of M segments shows that the Gn protein is more variable than the carboxy terminal protein (Gc). As for the S segment, the Thailand virus shows a similar amino acid sequence of the Gn and Gc proteins as the Jurong virus. Unfortunately, due to limited sequence information available, with regards to the Cambodian and Serang hantavirus strains, a proper comparison of the M segment sequences could not be performed. However, after analyzing the phylogenetic relationships, it is evident that the Serang, Cambodian, Thailand and the Jurong viruses are genetically related and form a distinct phylogroup of hantaviruses. As these genetically related viruses are harboured by different rodent species, this might suggest the possibility of a host-switch event [13,30].

Even though the trapping sites were scattered over most of the Singapore island, all PCR positive *R. tanezumi *were trapped at the same location, a shipyard in the Jurong area. However, no such clustering of hantavirus PCR positive rats to specific locations was observed among the 21 hantavirus RNA positive *R. norvegicus *carrying the Seoul Singapore virus.

In conclusion, by genetic identification of the different rodent hosts, sequence analysis of the corresponding hantaviruses nucleotide sequences in context with geographical distribution we are able to learn more of ancestral viruses, virus evolution, host-switch events and virus migration routes. Other interesting question, which regards to the genetic diversity of hantaviruses in Southeast Asia is the impact on human health. The pathogenicity of hantaviruses of *B. indica *or *R. tanezumi *is still unclear and further studies of epidemiological and epizootiological studies are required. Work is underway to evaluate the significance of our findings in public health context by establishing the seroprevalence of rodent borne diseases in Singapore human population.

## Competing interests

The authors declare that they have no competing interests.

## Authors' contributions

PJ participated in the study design, participated in fieldwork, preparation and analysis of rodent samples, performed (phylo)genetic analyses and contributed to writing of the manuscript. GY participated in the study design, fieldwork, preparation and analysis of rodent samples and contributed to writing of the manuscript, HTL and CCS participated in fieldwork preparation and analysis of rodent samples. RK performed virus isolation, RNA preparation, sequencing, cloning and writing. LCN participated in the study design, fieldwork and coordination, and contributed to writing. GB participated in the coordination of virus characterization, sequencing and drafting of the manuscript. All authors read and approved the final manuscript.

## Supplementary Material

Additional file 1 **Figure S1 - Rodent identification**. The rodents from Singapore's wild rodent population were identified through PCR based amplification and sequencing of *cytochrome b *gene fragments, as described in Materials and Methods. The obtained sequences (red text), along with rodent sequences found in public databases were used for phylogenetic-based species identification and a few clades of interest are high-lighted. Rodents belonging to this study are indicated by arrows. Obtained rodent sequences are available from the authors or accessible from GenBank (GQ274946 - GQ274949). Click here for file

Additional file 2 **Table S1 - Sequence pair distances**. S segment: (1) Jurong TJK/06 (This study); (2) Serang virus (AM998808); (3) Camb117 and Camb132 (AJ427511.1); (4) Thailand virus (Nakhon Ratchasima/Bi0017/2004) (AM397664.1); (5) Seoul Singapore (This study); (6) Seoul SR11 (M34881.1); (7) Hantaan virus (AB027111.1); (8) DOBV, strain Dobrava-Belgrade (L41916.1). The nucleotide and the amino acid sequence identity comparisons were made using the complete open reading frames of the S-segment except for Cambodia (nt 329-961) or Serang virus (nt 1-815). Comparisons between the partial sequences of Serang virus and Cambodian sequences (2) and (3) were made using the 486 nt overlapping coding region. M segment: (1) Jurong TJK/06 (This study); (2) Serang virus (AM998807); (3) Thailand virus L08756); (4) Seoul Singapore (This study); (5) Seoul virus (NC_005237.1; (6) Hantavirus Gou3 (AF145977); (7) Hantaan virus (NC_005219); (8) Dobrava-Belgrade virus (L33685). The nucleotide and the amino acid sequence identity comparisons were made using the complete open reading frames of the M-segment except for the Serang virus when the nucleotides 1990-2332 were used. Click here for file
