# Supplementary material for: Molecular characterization of two hantavirus strains from different rattus species in Singapore
Source: Virol J. 2010 Jan 22;7:15. doi: 10.1186/1743-422X-7-15 (PMC2823670; doi:10.1186/1743-422X-7-15)
Supplement: Additional file 1 — Figure S1 - Rodent identification. The rodents from Singapore's wild rodent population were identified through PCR based amplification and sequencing of cytochrome b gene fragments, as described in Materials and Methods. The obtained sequences (red text), along with rodent sequences found in public databases were used for phylogenetic-based species identification and a few clades of interest are high-lighted. Rodents belonging to this study are indicated by arrows. Obtained rodent sequences are available from the authors or accessible from GenBank (GQ274946 - GQ274949). [file 1743-422X-7-15-S1.PPT]

## Slide 1
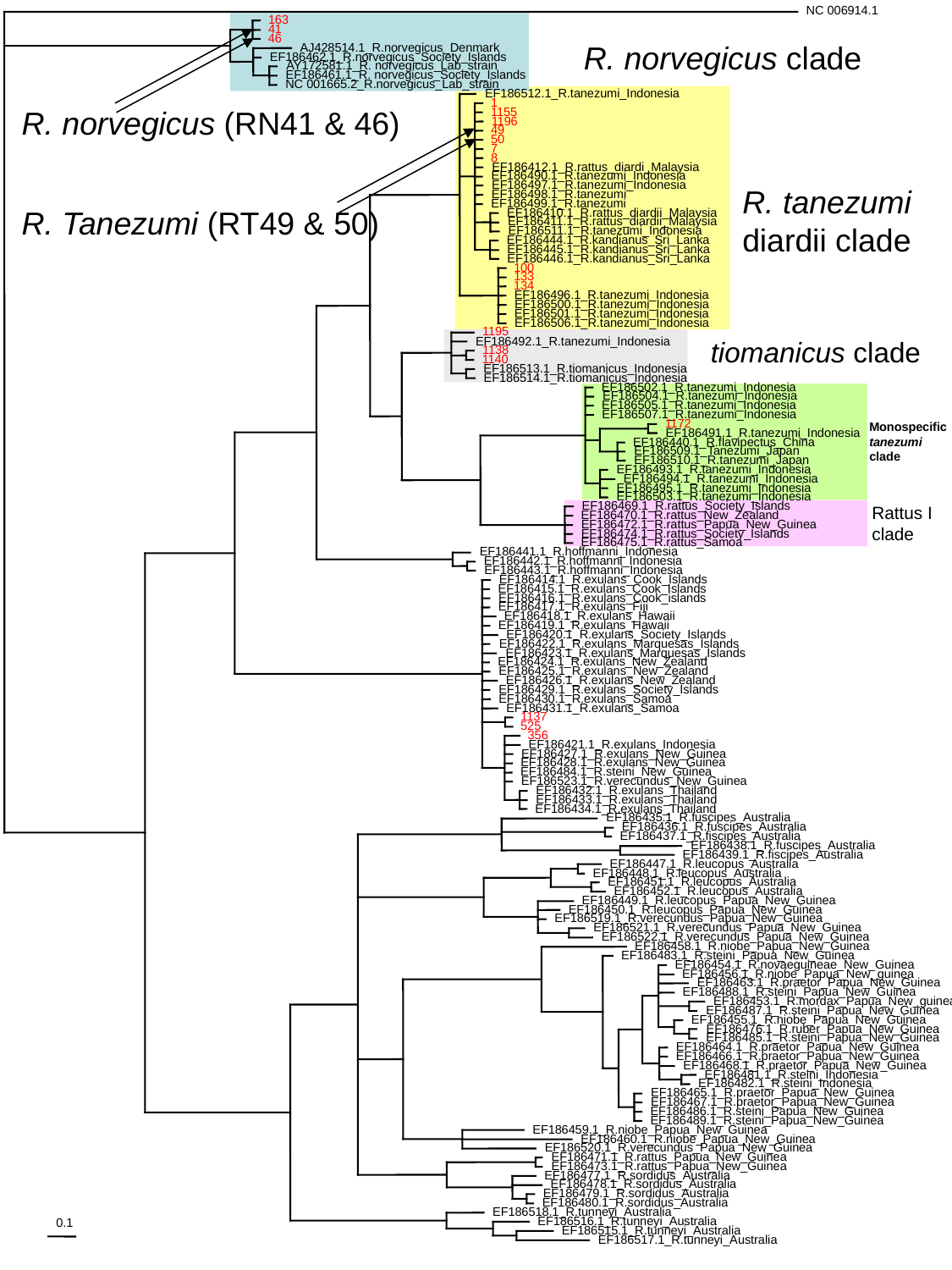

NC 006914.1
163
41
46
AJ428514.1_R.norvegicus_Denmark
EF186462.1_R.norvegicus_Society_Islands
AY172581.1_R. norvegicus_Lab_strain
EF186461.1_R. norvegicus_Society_Islands
NC 001665.2_R.norvegicus_Lab_strain
EF186512.1_R.tanezumi_Indonesia
1
1155
1196
49
50
7
8
EF186412.1_R.rattus_diardi_Malaysia
EF186490.1_R.tanezumi_Indonesia
EF186497.1_R.tanezumi_Indonesia
EF186498.1_R.tanezumi
EF186499.1_R.tanezumi
EF186410.1_R.rattus_diardii_Malaysia
EF186411.1_R.rattus_diardii_Malaysia
EF186511.1_R.tanezumi_Indonesia
EF186444.1_R.kandianus_Sri_Lanka
EF186445.1_R.kandianus_Sri_Lanka
EF186446.1_R.kandianus_Sri_Lanka
100
133
134
EF186496.1_R.tanezumi_Indonesia
EF186500.1_R.tanezumi_Indonesia
EF186501.1_R.tanezumi_Indonesia
EF186506.1_R.tanezumi_Indonesia
1195
EF186492.1_R.tanezumi_Indonesia
1138
1140
EF186513.1_R.tiomanicus_Indonesia
EF186514.1_R.tiomanicus_Indonesia
EF186502.1_R.tanezumi_Indonesia
EF186504.1_R.tanezumi_Indonesia
EF186505.1_R.tanezumi_Indonesia
EF186507.1_R.tanezumi_Indonesia
1172
EF186491.1_R.tanezumi_Indonesia
EF186440.1_R.flavipectus_China
EF186509.1_Tanezumi_Japan
EF186510.1_R.tanezumi_Japan
EF186493.1_R.tanezumi_Indonesia
EF186494.1_R.tanezumi_Indonesia
EF186495.1_R.tanezumi_Indonesia
EF186503.1_R.tanezumi_Indonesia
EF186469.1_R.rattus_Society_Islands
EF186470.1_R.rattus_New_Zealand
EF186472.1_R.rattus_Papua_New_Guinea
EF186474.1_R.rattus_Society_Islands
EF186475.1_R.rattus_Samoa
EF186441.1_R.hoffmanni_Indonesia
EF186442.1_R.hoffmanni_Indonesia
EF186443.1_R.hoffmanni_Indonesia
EF186414.1_R.exulans_Cook_Islands
EF186415.1_R.exulans_Cook_Islands
EF186416.1_R.exulans_Cook_islands
EF186417.1_R.exulans_Fiji
EF186418.1_R.exulans_Hawaii
EF186419.1_R.exulans_Hawaii
EF186420.1_R.exulans_Society_Islands
EF186422.1_R.exulans_Marquesas_Islands
EF186423.1_R.exulans_Marquesas_Islands
EF186424.1_R.exulans_New_Zealand
EF186425.1_R.exulans_New_Zealand
EF186426.1_R.exulans_New_Zealand
EF186429.1_R.exulans_Society_Islands
EF186430.1_R.exulans_Samoa
EF186431.1_R.exulans_Samoa
1137
525
356
EF186421.1_R.exulans_Indonesia
EF186427.1_R.exulans_New_Guinea
EF186428.1_R.exulans_New_Guinea
EF186484.1_R.steini_New_Guinea
EF186523.1_R.verecundus_New_Guinea
EF186432.1_R.exulans_Thailand
EF186433.1_R.exulans_Thailand
EF186434.1_R.exulans_Thailand
EF186435.1_R.fuscipes_Australia
EF186436.1_R.fuscipes_Australia
EF186437.1_R.fiscipes_Australia
EF186438.1_R.fuscipes_Australia
EF186439.1_R.fiscipes_Australia
EF186447.1_R.leucopus_Australia
EF186448.1_R.leucopus_Australia
EF186451.1_R.leucopus_Australia
EF186452.1_R.leucopus_Australia
EF186449.1_R.leucopus_Papua_New_Guinea
EF186450.1_R.leucopus_Papua_New_Guinea
EF186519.1_R.verecundus_Papua_New_Guinea
EF186521.1_R.verecundus_Papua_New_Guinea
EF186522.1_R.verecundus_Papua_New_Guinea
EF186458.1_R.niobe_Papua_New_Guinea
EF186483.1_R.steini_Papua_New_Guinea
EF186454.1_R.novaeguineae_New_Guinea
EF186456.1_R.niobe_Papua_New_guinea
EF186463.1_R.praetor_Papua_New_Guinea
EF186488.1_R.steini_Papua_New_Guinea
EF186453.1_R.mordax_Papua_New_guinea
EF186487.1_R.steini_Papua_New_Guinea
EF186455.1_R.niobe_Papua_New_Guinea
EF186476.1_R.ruber_Papua_New_Guinea
EF186485.1_R.steini_Papua_New_Guinea
EF186464.1_R.praetor_Papua_New_Guinea
EF186466.1_R.praetor_Papua_New_Guinea
EF186468.1_R.praetor_Papua_New_Guinea
EF186481.1_R.steini_Indonesia
EF186482.1_R.steini_Indonesia
EF186465.1_R.praetor_Papua_New_Guinea
EF186467.1_R.praetor_Papua_New_Guinea
EF186486.1_R.steini_Papua_New_Guinea
EF186489.1_R.steini_Papua_New_Guinea
EF186459.1_R.niobe_Papua_New_Guinea
EF186460.1_R.niobe_Papua_New_Guinea
EF186520.1_R.verecundus_Papua_New_Guinea
EF186471.1_R.rattus_Papua_New_Guinea
EF186473.1_R.rattus_Papua_New_Guinea
EF186477.1_R.sordidus_Australia
EF186478.1_R.sordidus_Australia
EF186479.1_R.sordidus_Australia
EF186480.1_R.sordidus_Australia
EF186518.1_R.tunneyi_Australia
EF186516.1_R.tunneyi_Australia
0.1
EF186515.1_R.tunneyi_Australia
EF186517.1_R.tunneyi_Australia
R. norvegicus clade
R. norvegicus (RN41 & 46)
R. tanezumi
diardii clade
R. Tanezumi (RT49 & 50)
tiomanicus clade
Monospecific
tanezumi
clade
Rattus I
clade
