# Supplementary material for: Molecular characterization of two hantavirus strains from different rattus species in Singapore
Source: Virol J. 2010 Jan 22;7:15. doi: 10.1186/1743-422X-7-15 (PMC2823670; doi:10.1186/1743-422X-7-15)
Supplement: Additional file 2 — Table S1 - Sequence pair distances. S segment: (1) Jurong TJK/06 (This study); (2) Serang virus (AM998808); (3) Camb117 and Camb132 (AJ427511.1); (4) Thailand virus (Nakhon Ratchasima/Bi0017/2004) (AM397664.1); (5) Seoul Singapore (This study); (6) Seoul SR11 (M34881.1); (7) Hantaan virus (AB027111.1); (8) DOBV, strain Dobrava-Belgrade (L41916.1). The nucleotide and the amino acid sequence identity comparisons were made using the complete open reading frames of the S-segment except for Cambodia (nt 329-961) or Serang virus (nt 1-815). Comparisons between the partial sequences of Serang virus and Cambodian sequences (2) and (3) were made using the 486 nt overlapping coding region. M segment: (1) Jurong TJK/06 (This study); (2) Serang virus (AM998807); (3) Thailand virus L08756); (4) Seoul Singapore (This study); (5) Seoul virus (NC_005237.1; (6) Hantavirus Gou3 (AF145977); (7) Hantaan virus (NC_005219); (8) Dobrava-Belgrade virus (L33685). The nucleotide and the amino acid sequence identity comparisons were made using the complete open reading frames of the M-segment except for the Serang virus when the nucleotides 1990-2332 were used. [file 1743-422X-7-15-S2.DOC]

**Table S1 - Sequence pair distances**

| ***S segment***  ***Nucleotide identity*** | | | | | | | | | |
| --- | --- | --- | --- | --- | --- | --- | --- | --- | --- |
| **Amino acid identity** |  | 1 | 2 | 3 | 4 | 5 | 6 | 7 | *8* |
|  | 1 |  | 95.0 | 85.8 | 83.6 | 77.8 | 77.6 | 75.0 | *75.6* |
|  | 2 | 99.6 |  | 85.4 | 84.2 | 73.0 | 72.7 | 73.0 | *75.2* |
|  | 3 | 98.2 | 99.2 |  | 83.1 | 77.1 | 76.6 | 73.0 | *72.8* |
|  | 4 | 96.7 | 96.7 | 98.8 |  | 76.8 | 77.1 | 74.7 | *73.7* |
|  | 5 | 87.0 | 86.8 | 78.2 | 86.5 |  | 97.1 | 74.6 | *74.3* |
|  | 6 | 86.0 | 85.7 | 78.2 | 86.3 | 97.9 |  | 74.7 | *74.3* |
|  | 7 | 83.5 | 82.4 | 75.8 | 83.0 | 82.1 | 81.2 |  | *74.1* |
|  | *8* | *82.1* | *80.5* | *76.4* | *83.2* | *81.2* | *79.8* | *82.8* |  |
| ***M segment***  ***Nucleotide identity*** | | | | | | | | | |
| **Amino acid identity** |  | 1 | 2 | 3 | 4 | 5 | 6 | 7 | *8* |
|  | 1 |  | 94.5 | 79.6 | 74.9 | 74.8 | 73.9 | 71.9 | *71.3* |
|  | 2 | 100 |  | 84.5 | 75.8 | 74.9 | 73.8 | 73.5 | *70.6* |
|  | 3 | 92.6 | 97.4 |  | 74.9 | 74.7 | 74.3 | 72.3 | *72.0* |
|  | 4 | 83.7 | 90.4 | 82.3 |  | 96.1 | 84.5 | 72.6 | *71.9* |
|  | 5 | 83.2 | 89.5 | 82.0 | 98.9 |  | 84.2 | 72.5 | *70.7* |
|  | 6 | 83.2 | 90.4 | 81.9 | 97.2 | 96.7 |  | 72.5 | *71.1* |
|  | 7 | 78.8 | 78.1 | 77.1 | 77.1 | 77.1 | 77.2 |  | *70.7* |
|  | *8* | *78.7* | *79.8* | *76.6* | *77.5* | *77.2* | *76.7* | *77.2* |  |
